# Lower Body Temperature Independently Predicts Delayed Cerebral Infarction in the Elderly With Ruptured Intracranial Aneurysm

**DOI:** 10.3389/fneur.2021.763471

**Published:** 2022-01-03

**Authors:** Hui Lin, Haojie Wang, Yawen Xu, Zhangya Lin, Dezhi Kang, Shufa Zheng, Peisen Yao

**Affiliations:** ^1^Department of Emergency, The First Affiliated Hospital, Fujian Medical University, Fuzhou, China; ^2^Department of Neurosurgery, Neurosurgery Research Institute, The First Affiliated Hospital, Fujian Medical University, Fuzhou, China; ^3^Fujian Key Laboratory of Precision Medicine for Cancer, The First Affiliated Hospital, Fujian Medical University, Fuzhou, China; ^4^Key Laboratory of Radiation Biology of Fujian Higher Education Institutions, The First Affiliated Hospital, Fujian Medical University, Fuzhou, China

**Keywords:** cerebral infarction, intracranial aneurysm, risk factor, diabetes, hypertension, body temperature

## Abstract

**Purpose:** To assess the correlation between admission body temperature and delayed cerebral infarction in elderly patients with ruptured intracranial aneurysm (IA).

**Methods:** Patients with ruptured IA diagnosed between 2012 and 2020 were retrospectively analyzed. Patients were divided into a non-infarction and an infarction group based on the presence of cerebral infarction after treatment. The demographic and clinical information of the patients was gathered. Outcomes at the 3-month follow-up were assessed using the modified Rankin Scale. Correlation between admission body temperature and cerebral infarction was assessed using Spearman's rank correlation coefficient. A receiver operating characteristic (ROC) curve was used to assess the specificity and sensitivity of admission body temperature to predict cerebral infarction.

**Results:** A total of 426 patients (142 men and 284 women) with ruptured IA were enrolled. Elderly patients with cerebral infarction (12.4%) had a lower body temperature at admission (*p* < 0.001), higher prevalence of hypertension and diabetes (*p* = 0.051 and *p* = 0.092, respectively), and higher rate of poor outcomes (*p* < 0.001). Admission body temperature was independently associated with cerebral infarction (odds ratio [OR] = 5.469, *p* < 0.001); however, hypertension (OR = 0.542, *p* = 0.056), diabetes (OR = 0.750, *p* = 0.465), and aneurysm size (OR = 0.959, *p* = 0.060) showed no association. An inverse correlation between admission body temperature and the incidence of cerebral infarction was observed (Spearman's *r* =−0.195, *p* < 0.001). An admission body temperature of 36.6°C was able to distinguish infarction and non-infarction patients. The area under the ROC curve was 0.669 (specificity, 64.15%; sensitivity, 81.50%; *p* < 0.001).

**Conclusions:** Lower body temperature at admission (≤36.6°C) is an independent predictor of delayed cerebral infarction in elderly patients who have undergone treatment for ruptured IA. Therefore, it could be a risk factor for adverse outcomes of IA.

## Introduction

Cerebral infarction following intracranial aneurysm (IA) rupture is a significant cause of unfavorable clinical outcomes (1). Several studies have shown that cerebral infarction is worsened by hyperthermia and alleviated by hypothermia (2–4). Particularly, it has been shown that hyperthermia is a predictor of in-hospital mortality and negatively affects 1-year survival probability for ischemic stroke but not 1-year mortality for hemorrhagic stroke (5, 6). Although it has been suggested that low body temperature is therapeutic, as it slows down the cerebral artery flow velocity leading to low perfusion pressure (7), this, in turn, may increase blood viscosity, promote erythrocyte aggregation and platelet microemboli, activate leukocytes, and reduce microcirculatory blood flow (8), ultimately resulting in arterial infarction. This notion is supported by several reports, including that of Naess et al., who have reported that low body temperature is associated with neurological worsening in patients with lacunar infarction (9). Similarly, Kvistad et al. reported that hypothermia within 6 h of symptom onset is associated with more severe neurological damage in the early phase of stroke (10).

Therefore, whether hypothermia aggravates the neurological function of stroke patients or reduces the risk of cerebral infarction following aneurysmal subarachnoid hemorrhage (SAH) still remains unclear (11, 12). The relationship between body temperature and stroke is controversial, specifically in elderly patients with IA who have undergone clipping or coiling. In fact, no reports have examined this topic. Therefore, this study aimed to investigate the relationship between body temperature at admission and cerebral infarction in elderly patients with ruptured IA. To elucidate this, we retrospectively analyzed the different clinical outcomes of elderly patients with or without cerebral infarction following IA treatment.

## Materials and Methods

### Patients

The Ethics Committee of the First Affiliated Hospital of Fujian Medical University approved the study and waived the requirement for written informed consent (approval number: MRCTA, ECFAH of FMU [2017] 079).

A total of 426 elderly patients with ruptured IA who underwent treatment between 2012 and 2020 were enrolled. The inclusion criteria were the following: (1) ruptured IA diagnosed by computed tomography angiography (CTA) and/or digital subtraction angiography (DSA) and (2) age ≥ 60 years. The exclusion criteria were the following: (1) prior history or presence of intracranial arteriovenous malformations, arteriovenous fistula, or moyamoya disease; (2) prior history of ischemic stroke; (3) presence of ongoing infections, such as pneumonia and sepsis; and (4) age <60 years. Patients who developed delayed cerebral infarction during hospitalization were also enrolled.

### Data Collection

The following demographic and clinical characteristics of patients with ruptured IA were gathered: age, sex, pulse rate, body temperature at admission (measured at the armpit), systolic blood pressure (SBP), diastolic blood pressure (DBP), medical history of hypertension and diabetes, Hunt-Hess (H-H) grade, Fisher grade, aneurysm location and size, treatment method, duration of temporary clipping (0, 0–5, 5–10, 10–15, >15 min), surgical time (duration between morbidity and surgery), laboratory data on admission (white blood cells, neutrophils, and lymphocytes), hemoglobin levels, coagulation parameters (prothrombin time, activated partial prothrombin time, international normalized ratio [INR], fibrinogen, D-dimer), season of admission (spring: March–May; summer: June–August; autumn: September–November; winter: December–February), and outcomes at the 3-month follow-up.

### Patient Treatment

Delayed cerebral infarction was diagnosed in the cases where new or worsening focal neurological deficits, such as hemiparesis, aphasia, or neglect, and decreased levels of consciousness were present. A decreased level of consciousness without focal neurological deficits was considered as a global or non-localized change. In the cases in which these critically ill patients were stable, computed tomography (CT) or magnetic resonance imaging (MRI) were performed. Specifically, in the cases in which CT scans could not detect delayed cerebral infarction, MRI was performed. The clinical symptom patterns of delayed cerebral infarction were also recorded, and the location of cerebral infarction was determined as previously described (13).

Treatment methods for IA included microsurgical clipping, endovascular coiling, or other conservative methods. Perioperative treatment was performed according to the Chinese guidelines for the management of aneurysmal SAH. All patients underwent CTA or DSA examination within 7 postoperative days to determine whether cerebral vasospasm or residual neck aneurysm had occurred. The 3-month outcome was assessed using the modified Rankin Scale (mRS). Scores of 0–2 were considered as good outcomes, whereas scores of 3–6 indicated poor outcomes.

### Data Analysis

Statistical analyses were performed using SPSS version 26.0 (IBM, Chicago, IL, USA). The homogeneity of variance was evaluated. Student's *t*-test or a one-way analysis of variance (ANOVA) was employed to compare continuous variables, while the chi-squared test (χ^2^ test) or Fisher's exact test was utilized to evaluate qualitative variables. Variables that achieved a significance of *p* < 0.10 in the univariate analysis were employed in the multivariable analysis. The correlation between body temperature and cerebral infarction was assessed using Spearman's rank correlation coefficient. The difference in body temperature between the positive outcome group (mRS scores 0–2) and poor outcome group (mRS scores 3–6) was computed with the Mann–Whitney U test. Statistical significance was set at *p* < 0.05. A receiver operating characteristic (ROC) curve was used to assess the specificity and sensitivity of admission body temperature for predicting cerebral infarction in elderly patients with ruptured IA.

## Results

### Demographic and Clinical Characteristics

**Figure 1:**
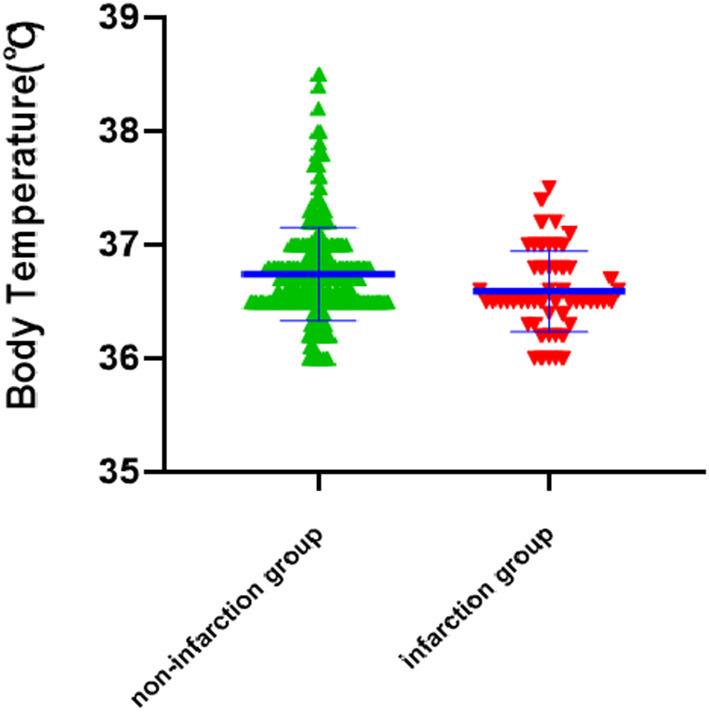
The scatterplot graph for the correlation analysis of body temperature and cerebral infarction (*P* < 0.001).

The medical records of 426 patients (142 men and 284 women) with ruptured IA were retrospectively analyzed. Patients were divided into an infarction group (*n* = 53) or a non-infarction group (*n* = 373) based on the presence of cerebral infarction following IA treatment. Table 1 summarizes the demographic and clinical data of the patients. The infarction group had a lower body temperature at admission (infarction vs. non-infarction: 36.67 ± 0.38°C vs. 36.89 ± 0.37; *p* < 0.001) (Figure 1), a lower prevalence of hypertension (33.9% vs. 48.3%; *p* = 0.051), a higher prevalence of diabetes (20.8% vs. 12.3%; *p* = 0.092), and a higher rate of poor outcomes (32.1% vs. 12.1%; *p* < 0.001). No other statistical differences in the analyzed variables were detected between the two groups (*p* > 0.05).

**Table 1:** Characteristics of infarction and non-infarction groups.

**Characteristics**	**Infarction group**	**Non-infarction group**	***p*** **-value**
	**(Group I, ***n*** = 53)**	**(Group II, ***n*** = 373)**	
Age(years)	66.64 ± 3.89	65.99 ± 5.19	0.377
**Gender**			0.177
Female	31 (58.5%)	253 (67.8%)	
Male	22 (41.5%)	120 (32.2%)	
Pulse	79.74 ± 14.14	77.23 ± 16.94	0.305
Body temperature (°C)	36.67 ± 0.38	36.89 ± 0.37	0.000
SBP, mmHg	144.08 ± 25.92	143.44 ± 24.87	0.862
DBP, mmHg	84.75 ± 11.73	82.07 ± 13.78	0.178
**Hypertension**			0.051
No	35 (66.1%)	193 (51.7%)	
Yes	18 (33.9%)	180 (48.3%)	
**Diabetes**			0.092
No	42 (79.2%)	327 (87.7%)	
Yes	11 (20.8%)	46 (12.3%)	
**Hunt-Hess grade**			0.228
Grade I	8 (15.1%)	64 (17.2%)	
Grade II	20 (37.7%)	137 (36.7%)	
Grade III	12 (22.6%)	118 (31.6%)	
Grade IV	10 (18.9%)	34 (9.1%)	
Grade V	3 (5.7%)	20 (5.4%)	
**Fisher grade**			0.646
Grade 0	10 (18.8%)	62 (16.6%)	
Grade 1	4 (7.5%)	49 (13.1%)	
Grade 2	14 (26.4%)	115 (30.8%)	
Grade 3	12 (22.6%)	64 (17.2%)	
Grade 4	13 (24.5%)	83 (22.3%)	
**Aneurysm location**			0.722
ACA	1 (1.9%)	19 (5.1%)	
ACoA	12 (22.6%)	91 (24.4%)	
ICA	11 (20.8%)	68 (18.2%)	
MCA	14 (26.4%)	71 (19.0%)	
PCoA	12 (22.6%)	98 (26.3%)	
others	3 (5.7%)	26 (7.0%)	
Aneurysm size	7.67 ± 6.93	6.12 ± 5.44	0.061
**Treatment methods**			0.591
Microsurgical clipping	41 (77.3%)	301 (80.7%)	
Endovascular coiling	5 (9.4%)	39 (10.5%)	
Conservative treatment	7 (13.2%)	33 (8.8%)	
**Duration of temporary clip**			0.847
0 min	8 (19.5%)	48 (15.9%)	
0–5 min	21 (51.2%)	174 (57.8%)	
5–10 min	8 (19.5%)	61 (20.3%)	
10–15 min	3 (7.3%)	14 (4.7%)	
>15 min	1 (2.4%)	4 (1.3%)	
**Surgical time**			0.972
<3days	28 (52.8%)	198 (53.1%)	
>3days	25 (47.2%)	175 (46.9%)	
Serum leukocyte count (*109/L)	9.81 ± 3.88	9.30 ± 3.99	0.380
Neutrophil	7.62 ± 3.79	8.76 ± 10.11	0.317
Lymphocyte	1.36 ± 0.64	1.43 ± 1.89	0.791
Hemoglobin, g/L	127.09 ± 14.24	124.66 ± 17.63	0.337
**Coagulation parameters**		
Prothrombin time(s)	11.86 ± 1.11	11.91 ± 0.75	0.686
Activated partial prothrombin time(s)	29.71 ± 6.06	29.46 ± 6.38	0.793
INR	0.98 ± 0.12	1.01 ± 0.10	0.162
Fib fibrinogen(g/l)	2.98 ± 0.96	3.17 ± 1.64	0.416
D-dimer (ug/mL)	2.48 ± 1.98	1.99 ± 2.15	0.201
**Seasons**			0.348
Spring (March–May)	13 (24.5%)	96 (25.7%)	
Summer (June–August)	9 (17.0%)	93 (24.9%)	
Autumn (September–November)	14 (26.4%)	102 (27.3%)	
Winter (December–February)	17 (32.1%)	82 (22.0%)	
**mRS**			0.000
0–2	36 (67.9%)	328 (87.9%)	
3–6	17 (32.1%)	45 (12.1%)	

### Correlation Between Admission Body Temperature and Cerebral Infarction

**Figure 2:**
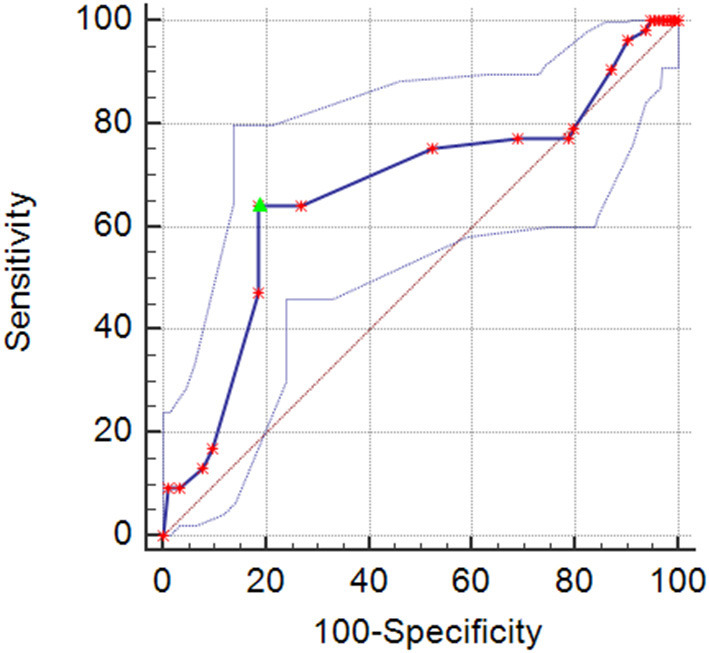
The receiver operating characteristic (ROC) curve for the correlation between body temperature and the occurrence of cerebral infarction in elderly IA patients (the upper and lower dotted curves are the 95% confidence intervals), area under curve 0.669(95% confidence interval [CI], 0.622–0.714; *p* < 0.001). The cutoff = 36.6°C (sensitivity = 64.15%, specificity = 81.50%).

The results of the multivariable logistic regression analysis showed that admission body temperature was independently associated with cerebral infarction in elderly patients with IA (odds ratio [OR] = 5.469; 95% confidence interval [CI] = 2.202–13.581; *p* < 0.001). However, hypertension (OR = 0.542, 95% CI = 0.289–1.015; *p* = 0.056), diabetes (OR = 0.750, 95% CI: 0.347–1.623; *p* = 0.465), and aneurysm size (OR = 0.959, 95% CI = 0.917–1.002; *p* = 0.060) showed no correlation with cerebral infarction (Table 2). There was an inverse correlation between admission body temperature and the incidence of cerebral infarction (Spearman's *r* = −0.195, *p* < 0.001). Figure 2 shows the ROC curve for the specificity and sensitivity of admission body temperature in predicting cerebral infarction. An admission body temperature of 36.6°C was able to distinguish infarction and non-infarction patients. The area under the ROC curve was 0.669 (specificity: 64.15%; sensitivity: 81.50%; *p* < 0.001) (Figure 2).

**Table 2:** Independent risk factors associated with cerebral infarction.

	**Unadjusted**			**Adjusted**		
**Parameter**	**OR**	**OR (95%CI)**	***P*** **-value**	**OR**	**OR (95%CI)**	***P*** **-value**
Body Temperature	5.561	2.272–13.611	0.000	5.469	2.202–13.581	0.000
Hypertension	0.551	0.302–1.008	0.053	0.542	0.289–1.015	0.056
Diabetes	0.537	0.258–1.117	0.096	0.750	0.347–1.623	0.465
Aneurysm size	0.961	0.921–1.003	0.066	0.959	0.917–1.002	0.060

### Relationship Between Clinical Manifestation and Imaging Findings

The relationship between the symptoms and imaging findings of patients with cerebral infarction is shown in Table 3. Clinical manifestations included symptomatic cerebral infarction (*n* = 43, 81.1%), focal neurological deficits (*n* = 26, 49.1%), and global or non-localized changes (*n* = 17, 32.1%). Cerebral infarctions involved single cortical (*n* = 16, 30.2%), single deep (*n* = 16, 30.2%), multiple cortical (*n* = 10, 18.9%), multiple deep (*n* = 2, 3.8%), and multiple combined cortical and deep territories (*n* = 8, 15.1%). Asymptomatic cerebral infarction frequently occurred in patients with single deep infarction. In addition, cerebral infarction more frequently involved a single cortical or a single deep territory.

**Table 3:** Clinical manifestations of delayed cerebral infarction according to the pattern in the infarction group.

**Pattern of delayed cerebral infarction**	**Clinical manifestation**			
	**Asymptomatic (***n***%)**	**Focal neurological deficits (***n***%)**	**Global or non-localizing (***n***%)**	**Total (***n***%)**
Single cortical	2 (3.8%)	12 (22.6%)	2 (3.8%)	16 (30.2%)
Single deep	6 (11.3%)	6 (11.3%)	5 (9.4%)	17 (32.1%)
Multiple cortical	1 (1.9%)	4 (7.5%)	5 (9.4%)	10 (18.9%)
Multiple deep	0 (0%)	1 (1.9%)	1 (1.9%)	2 (3.8%)
Multiple combined cortical and deep	0 (0%)	3 (5.7%)	5 (9.4%)	8 (15.1%)
Total	9 (17.0%)	26 (49.1%)	18 (33.9%)	53 (100.0%)

*Hemiparesis, aphasia, or neglect was considered as focal neurological deficits. Decreased level of consciousness without focal neurological deficits was considered as global or non-localizing*.

## Discussion

In this study, we explored the association between admission body temperature and cerebral infarction in elderly patients with ruptured IA. Our results demonstrated that patients with cerebral infarction had a lower body temperature at admission, higher prevalence of hypertension and diabetes, and a higher rate of poor outcomes than patients without infarction. In addition, multivariate logistic regression analysis revealed that body temperature is independently associated with cerebral infarction, and a body temperature of 36.6°C is the optimal cutoff value to distinguish infarction and non-infarction patients.

Rupture of IAs is one of the most common causes of stroke and results in high mortality and increased risk of disability. Disturbances in normal brain metabolism are known to play a role in the prognosis of SAH (14), and several cerebral metabolites, including lactate (15) and pyruvate (16), are considered predictive biomarkers of aneurysmal SAH outcomes. Several reports showed that hypothermia is therapeutic, as it reduces intracranial pressure and improves functional recovery (7, 17), while hypometabolism could prevent or reduce secondary brain injury following SAH (18). It is possible that hypothermia suppresses brain metabolism and protects the brain tissue from potentially injurious conditions in which brain metabolism is disturbed. However, the detailed mechanism by which hypothermia exerts its protective effects on brain tissues remains unclear. Conversely, recent studies showed that moderate hypothermia might not be beneficial for stroke patients (19), as it did not reduce the likelihood of death or disability in patients with neonatal encephalopathy, but rather, it significantly increased the risk of death (20). Rahmig et al. (19) found that, in surgically treated patients with middle carotid artery (MCA) infarction, the risk of death was lower for individuals with high body temperature than for those with low body temperature. The discrepancies observed in the abovementioned findings may be explained by the fact that low body temperature and therapeutic hypothermia may be different conditions. In fact, the former is due to reduced heat production by the body, which can arise from any number of circumstances, while therapeutic or iatrogenic hypothermia is an externally controlled reduction in body temperature. Therefore, in cerebral ischemic conditions, the mechanisms underlying the response to therapeutic hypothermia and low body temperature might be different. However, such mechanisms remain unclear.

Evidence suggests that therapeutic hypothermia decreases cerebral artery flow velocity (7). Low body temperature could operate through this same mechanism. Low perfusion pressure may promote the ability of blood viscosity, red cell aggregation, platelet microemboli, and activated leucocytes to reduce microcirculatory blood flow (8), ultimately leading to artery infarction. This hypothesis is supported by the findings of Starnoni et al., who showed that delayed cerebral ischemia is associated with reduced cerebral blood flow (21). More importantly, the temporary blocking of the parent artery during cerebral aneurysm clipping is likely to aggravate the ischemic cerebral injury induced by decreased cerebral artery flow. In older adults, the observed low basal metabolic rate is also caused by low body temperature. Studies suggest that glucose hypometabolism might be an early risk factor for cerebral infarction (8), indicating that low temperature-induced hypometabolism could predict cerebral infarction in elderly patients with IA. However, the role of glucose hypometabolism needs to be further studied.

Our research has several limitations. This was a retrospective study with a small sample size, and relevant metabolic factors, such as the basal metabolic rate, were not included. In addition, our study solely focuses on patients with ruptured IA; thus, our results cannot be generalized to cases of unruptured IA. Further research with a larger sample size is needed to conducted subgroup analyses and understand the specific mechanisms of action of hypothermia and low body temperature. In conclusion, lower admission body temperature (≤ 36.6°C) is an independent predictor of delayed cerebral infarction and therefore could be a risk factor for adverse outcomes in elderly patients with ruptured IA.

## Data Availability Statement

The original contributions presented in the study are included in the article/supplementary material, further inquiries can be directed to the corresponding author/s.

## Ethics Statement

The studies involving human participants were reviewed and approved by the Ethics Committee of the First Affiliated Hospital of Fujian Medical University. Written informed consent for participation was not required for this study in accordance with the national legislation and the institutional requirements.

## Author Contributions

HL, HW, YX, and ZL: acquisition of data and critical revision of manuscript for intellectual content. DK, SZ, and PY: study concept and design. SZ and PY: analysis and interpretation of data and study supervision. All authors contributed to the article and approved the submitted version.

## Funding

This work was supported by key Clinical Specialty Discipline Construction Program of Fujian, P.R.C, major project of Fujian Provincial Department of Science and Technology (No. 2014YZ0003 and No. 2014YZ01 to DK), the Young and Middle-aged Backbone Key Research Project of National Health and Family Planning Commission of Fujian Province (No. 2017-ZQN-46 to PY), Natural Science Funding of Fujian Province (No. 2018J01175 to PY and No. 2018J01176 to SZ) and Natural Science Funding of China (No. 81802492 to PY).

## Conflict of Interest

The authors declare that the research was conducted in the absence of any commercial or financial relationships that could be construed as a potential conflict of interest.

## Publisher's Note

All claims expressed in this article are solely those of the authors and do not necessarily represent those of their affiliated organizations, or those of the publisher, the editors and the reviewers. Any product that may be evaluated in this article, or claim that may be made by its manufacturer, is not guaranteed or endorsed by the publisher.
